# Mixed adenoneuroendocrine carcinoma of the gallbladder: a case report and review of the literature

**DOI:** 10.3389/fonc.2025.1678082

**Published:** 2026-02-20

**Authors:** Yang Du, Jiangnan Yang, Deyuan Fu

**Affiliations:** 1 Department of General Surgery, Clinical Medical College, Yangzhou University, Yangzhou, China; 2 Department of Basic Medicine, Medical College of Hunan Normal University, Changsha, China; 3 Department of Thyroid and Breast Surgery, Northern Jiangsu People’s Hospital Affiliated to Yangzhou University, Yangzhou, Jiangsu, China

**Keywords:** gallbladder, mixed adenoneuroendocrine carcinoma, MANEC, neuroendocrine carcinoma, rare malignant tumor, GB-NEC

## Abstract

Neuroendocrine carcinoma (NEC) is a rare malignant tumor of the gallbladder (GB) and is extremely rare in medical practice. Related case reports and in-depth studies are extremely limited, resulting in an insufficient understanding of the disease. This article reports a case of a 60-year-old female patient who complained of right upper abdominal pain for 20 days. Subsequent examinations confirmed that it was mixed adenoneuroendocrine carcinoma of the gallbladder (GB-MANEC), of which the neuroendocrine part accounted for approximately 70%, and was defined as a poorly differentiated NEC. The Ki-67 index reached 80%, indicating highly aggressive behavior. Postoperative adjuvant chemotherapy was administered (six cycles of etoposide combined with cisplatin). Follow-up through December 2024 showed no signs of tumor recurrence, with a disease-free survival period of 12 months. Currently, the preferred treatment strategy for GB-NEC is radical surgery. Therefore, in this case, radical resection combined with chemotherapy became an effective treatment.

## Introduction

Neuroendocrine neoplasms (NENs) are rare tumors originating from neuroendocrine cells, formerly known as Kulchitsky cells or argyrophilic cells, and can arise in various body sites, including the gastrointestinal tract, lungs, cervix, bladder, prostate, and pancreas. They are most common in the gastrointestinal and respiratory tracts. Gallbladder neuroendocrine neoplasms (GB-NENs) are clinically rare, accounting for 0.5% of all NENs and 2.1% of all gallbladder tumors according to the US Surveillance, Epidemiology, and End Results (SEER) database (1). Neuroendocrine carcinoma of the gallbladder (GB-NEC), a subset of GB-NENs, has an even lower incidence. GB-NENs are more prevalent in women, with a peak occurrence between 50 and 60 years and a male-to-female ratio of approximately 1:1.6 (2).

Due to the rarity of GB-NEC, there is a lack of large-scale clinical research data, and most research data are derived from case reports and single-center series studies. The treatment of the disease still faces ongoing challenges, and new therapies are emerging, such as immunotherapy, which has achieved complete remission in metastatic cases (3, 4). This article reports a case of GB-NEC and reviews the literature.

## Case presentation

**Figure 1:**
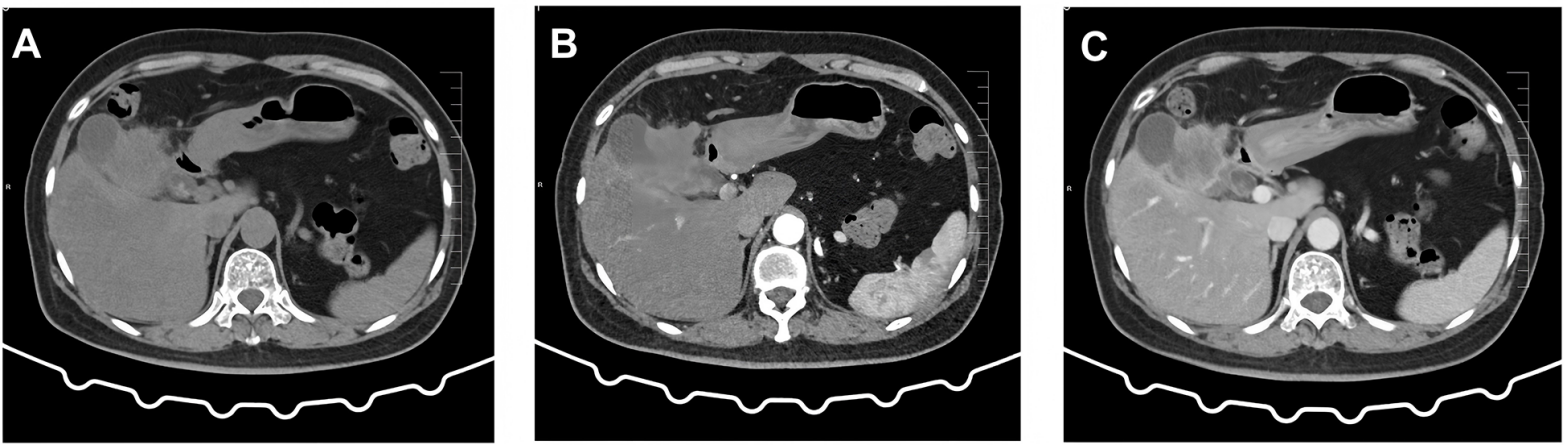
Preoperative abdominal CT findings. **(A)** Conventional scan reveals a low-density mass with indistinct borders in the gallbladder region. **(B)** Arterial phase scan shows heterogeneous enhancement in the surrounding area. **(C)** Venous phase scan demonstrates persistent but slightly diminished enhancement. The mass invades adjacent hepatic parenchyma (segments IVb and V), suggesting a localized gallbladder malignancy.

**Figure 2:**
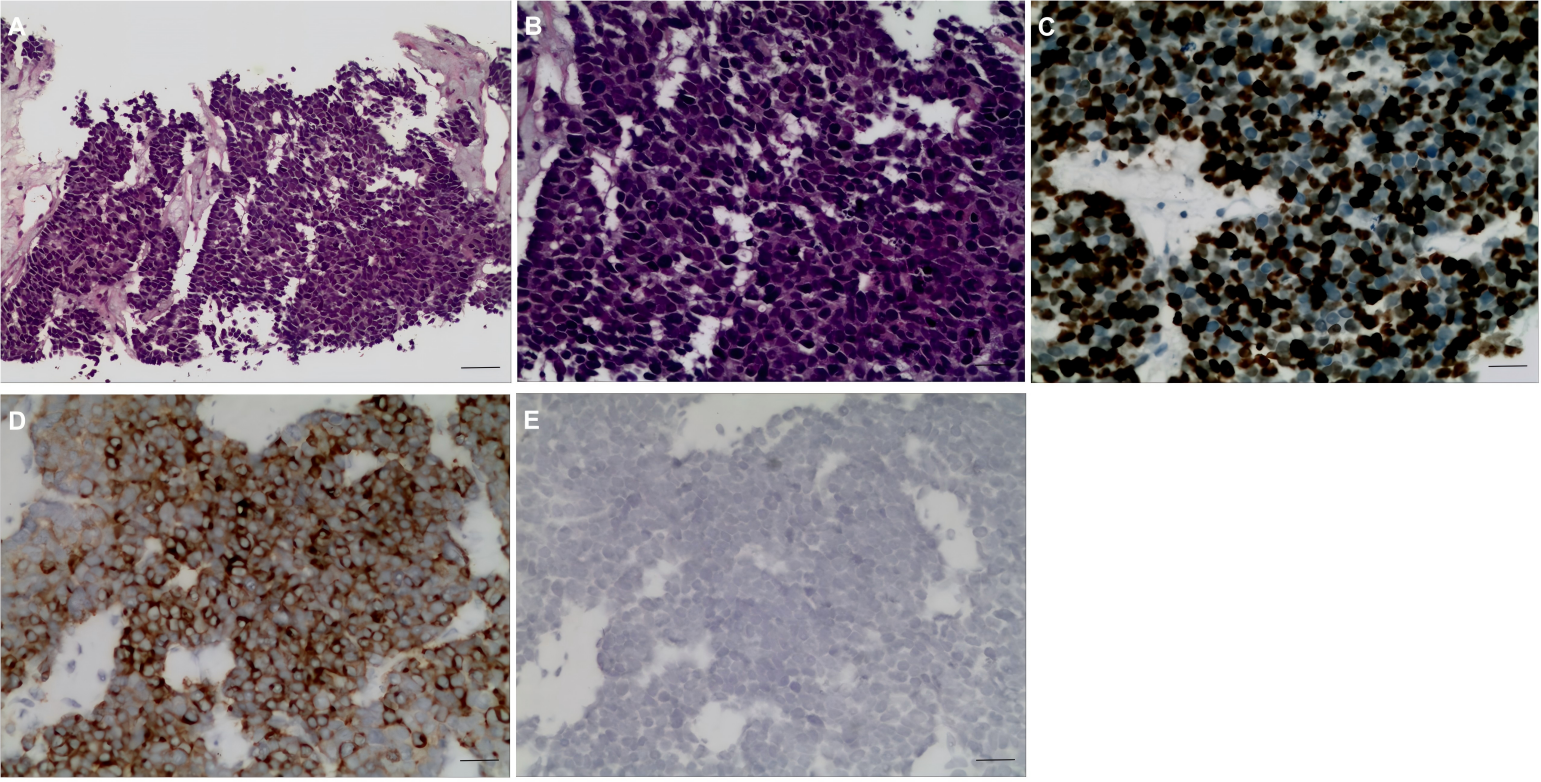
Pathological H&E staining and immunohistochemical results. **(A, B)** GB-NEC pathological map (H&E, ×100, ×200). **(C–E)** Immunohistochemical map. **(C)** Ki-67 (+80%), ×200. **(D)** Syn (+) ×200. **(E)** CgA (+), ×200. GB-NEC, neuroendocrine carcinoma of the gallbladder; Syn, synaptophysin; CgA, chromogranin.

A 60-year-old female patient was admitted to the hospital on December 06, 2023, due to “upper abdominal pain for 20 days”. She was previously in good health. Physical examination revealed epigastric tenderness with rebound tenderness, and Murphy’s sign was positive. Laboratory examination showed that carbohydrate antigen 19-9 (CA19-9), carbohydrate antigen 125 (CA125), carcinoembryonic antigen (CEA), alpha-fetoprotein (AFP), etc., were normal. Preoperative abdominal CT scan and enhancement are shown in **Figure 1**. Preoperative abdominal CT with plain and contrast-enhanced scans (**Figure 1**) revealed an irregular mass lesion measuring approximately 3.8 × 3.0 cm at the gallbladder site. On plain scan, it appeared slightly hypodense with ill-defined margins. During the arterial phase of contrast enhancement, it demonstrated heterogeneous, marked enhancement. In the venous phase, enhancement persisted but showed slight attenuation. It was accompanied by irregular thickening of the gallbladder wall and infiltrative growth into adjacent hepatic parenchyma (segments IVb and V). Mild dilatation of intrahepatic bile ducts was noted. Imaging features suggested a highly malignant gallbladder tumor, consistent with gallbladder carcinoma, with possible local liver involvement. Main diagnosis was as follows: 1) gallbladder space-occupying: gallbladder cancer? On December 10, 2023, the patient underwent a radical laparoscopic cholecystectomy for gallbladder cancer. The procedure included radical cholecystectomy for gallbladder cancer + segmental resection of segments IVb and V of the liver + lymph node dissection of the hepatoduodenal ligament + exploration of the common bile duct + T-tube drainage. The gallbladder fundus exhibited a grayish-white cut surface with a firm, hard texture. The lesion measured approximately 3.0 × 3.3 cm. Twelve lymph nodes were resected and submitted for examination during the procedure. Pathological examination showed the following: (gallbladder + liver segments 4, 5, 6 + hilar lymph nodes) gallbladder-mixed adenoneuroendocrine carcinomas, of which neuroendocrine carcinomas accounted for approximately 70%, exocrine carcinomas accounted for approximately 30%, and exocrine carcinomas accounted for approximately 30%. Approximately 15% of the carcinomas were moderately differentiated tubular adenocarcinomas, and 15% were moderately differentiated squamous cell carcinomas, which invaded the whole thickness of the gallbladder wall and invaded the serosal surface. Nerve invasion, multifocal hemorrhage, necrosis, and lymph nodes (0/12) were observed. Immunohistochemical results were as follows: NEC components Ki-67 (+80%), p63 (−), CK5/6 (+), CK7 (−), CK20 (+), Villin (−), CDX2 (−), mutation synaptophysin (Syn) (+), neuron-specific enolase (NSE) (−), CD56 (+), CK (pan) (−), and CD34 blood vessel (+), as shown in **Figure 2**. Final pathological staging (American Joint Committee on Cancer (AJCC) 8th edition) was pT3N0M0, Stage III A. The tumor invaded the entire wall of the gallbladder and penetrated the serosa, extending into adjacent liver parenchyma (segments IVb and V), but the resection margins were negative (R0 resection). All 12 lymph nodes in the hepatoduodenal ligament were free of metastasis (0/12). There was no evidence of distant metastasis on intraoperative examination or imaging studies. The patient received six cycles of etoposide plus cisplatin (EP) adjuvant chemotherapy (January–May 2024). Follow-up protocol included clinical evaluation (physical exam and symptom assessment) every 3 months, laboratory tests (Complete Blood Count (CBC), liver/kidney function, CA19-9, CEA, and NSE), and imaging (contrast-enhanced abdominal/pelvic CT + chest CT). As of December 2024 (12 months after operation), four follow-ups were completed with no evidence of recurrence or metastasis. Current disease-free survival (DFS) was 12 months with good performance status.

## Discussion

The most common primary sites of neuroendocrine tumors (NETs) vary slightly by region and study, but generally, they are mainly in the gastrointestinal tract (especially the small intestine and rectum) and the lungs (5). Among them, some epidemiological surveys have shown that the lungs account for approximately 25%, and the small intestine accounts for approximately 18%, while in North American and Asian data, the small intestine, rectum, and pancreas often rank at the top (6).

The 2022 WHO classification (7) categorizes neuroendocrine tumors into two main groups: well-differentiated NETs (G1–G3) and poorly differentiated NECs. Due to their high malignancy and rapid progression, most patients with GB-NEC present with lymph node and liver metastases at diagnosis, resulting in a poor prognosis. In this case, the Ki-67 index reached 80%, with a large cell morphology and marked necrosis, confirming the diagnosis of NEC. However, NETs represent a distinct category of neuroendocrine neoplasms, classified into three grades based on cellular differentiation: Grade 1 (mitotic count <2 and Ki-67 index <2%), Grade 2 (mitotic count 2–20 and Ki-67 index 3%–20%), and Grade 3 (mitotic count >20 and Ki-67 index >20%). Grade 1 tumors exhibit low malignancy with no significant early metastasis and generally carry a favorable prognosis.

The clinical manifestations of GB-NECs have no obvious specificity. Right upper quadrant pain is the most common initial symptom, followed by jaundice and emaciation. Since these symptoms are similar to the general clinical manifestations of gallbladder stones, cholecystitis, or gallbladder cancer, it is difficult to judge from preoperative auxiliary examinations, such as B-ultrasound, CT, and MRI. Less than 1% of all patients with GB-NECs had carcinoid syndrome (8).

Common examination methods for GB-NECs include tumor markers, abdominal color Doppler ultrasound, CT, and MRI. However, the results of tumor markers such as CA19–9 and CA125 are often negative, and ultrasonography can only find thickening of the gallbladder wall and raised lesions of the gallbladder wall, etc. CT and MRI examinations have difficulty detecting GB-NECs and differentiating them from other gallbladder tumors. In short, preoperative imaging examinations are only helpful for preliminary judgment of the source and nature of the tumor, but cannot confirm the type of tumor. The diagnosis of GB-NEC depends on postoperative pathological immunohistochemical staining, which is of great significance for the diagnosis and grading of tumors. The pathological type of tumor is an important factor in determining the prognosis of GB-NECs (9). Chromogranin (CgA), Syn, and other indicators have a high positive rate in NEC, which is also the main basis for the diagnosis of NEC.

Surgery is the first choice and the most effective treatment. The surgical methods mainly include simple cholecystectomy, cholecystectomy plus partial hepatectomy, and lymph node dissection. For gallbladder cancer *in situ* (cT1N0M0) or gallbladder cancer only invading the mucosa, submucosa, or muscular layer, cholecystectomy alone is feasible. For advanced patients without distant metastasis, cholecystectomy plus partial hepatectomy and lymph node dissection are feasible. This is due to the current lack of large-scale randomized controlled trials targeting GB-NEC. Presently, a retrospective cohort study published in 2025 (10) conducted a comparative analysis of postoperative survival between GB-NEC and adenocarcinoma following radical resection. The results showed that the 5-year overall survival (OS) rate after R0 resection in the NEC group was 38.7%, significantly superior to that in the R1/R2 resection group (p = 0.012), and showed no statistically significant difference compared with the adenocarcinoma group (HR 1.21, 95% CI 0.89–1.64). This study supports applying the same radical resection criteria for locally advanced but resectable NEC as for adenocarcinoma. In this case report, achieving R0 resection in a patient with pT3N0M0 staging aligns with the aforementioned evidence.

To further investigate the efficacy of R0 resection in GB-NEC, we retrospectively reviewed case reports indexed in PubMed from 2015 to 2025, identifying five cases of GB-NEC achieving R0 resection (**Table 1**). These cases predominantly involved middle-aged and elderly women, with large cell and mixed types being the most common, and clinical stage III being the most frequent. Surgery primarily involved radical cholecystectomy with hepatic wedge resection and lymph node dissection. Adjuvant chemotherapy (including platinum-based regimens) significantly improved prognosis. Follow-up revealed no early recurrence in most cases, although long-term survival remained influenced by Ki-67 index and lymph node status. This case (pT3N0M0, Ki-67 80%, six cycles of EP chemotherapy) aligns with the literature, with 12-month DFS supporting the aggressive strategy of R0 resection plus chemotherapy. However, further multicenter studies are needed to validate these findings.

**Table 1:** Summary of reported GB-NEC cases with R0 resection.

Case number	Country/region	Gender/age	NEC type	Stage	Surgery type	Margins	Adjuvant therapy	Outcome (follow-up status, recurrence, survival)	Reference
1	Japan	Female/70	Mixed (MANEC)	IIIB (T2bN1M0)	Radical cholecystectomy + partial hepatectomy + lymph node dissection	R0	Cisplatin + etoposide (4 cycles)	No recurrence; DFS 12 months	(15)
2	China	Male/53	NEC	III	Radical cholecystectomy + lymph node dissection	R0	Cisplatin + etoposide	Partial remission; OS 20 months, recurrence	(16)
3	USA	Female/73	Large-cell type	III	Radical cholecystectomy + hepatic wedge resection	R0	Cisplatin + etoposide (7 cycles)	No recurrence; DFS 36 months	(17)
4	Algeria	Female/68	Large-cell type	III	Cholecystectomy+ hepatic segment IVb/V resection + lymph node dissection	R0	Cisplatin + etoposide (4 cycles)	Recurrence (lymph node metastasis); OS 26 months	(18)
5	China	Male/52	Small-cell type	III	Radical cholecystectomy + lymph node dissection	R0	Cisplatin + etoposide (4 cycles)	Recurrence (liver metastasis); OS greater than 20 months	(19)

GB-NEC, neuroendocrine carcinoma of the gallbladder; MANEC, mixed adenoneuroendocrine carcinoma; DFS, disease-free survival; OS, overall survival.

Since GB-NECs are prone to systemic metastasis, postoperative adjuvant therapy plays an important role. For the adjuvant therapy after radical resection of GB-NECs, as there is no high-level evidence-based medical evidence, the principles of adjuvant therapy for pancreatic neuroendocrine carcinoma can be referred to. For patients with GB-NEC undergoing radical resection, postoperative chemotherapy is recommended even in the absence of high-risk factors such as lymph node metastasis or vascular tumor embolism (11). Platinum-based dual-agent chemotherapy (e.g., cisplatin or carboplatin + etoposide) is recommended (12). In this case, six cycles of the EP regimen were administered with good tolerability, and no recurrence was observed at 12 months of follow-up. The follow-up of this case was a combination of clinical, laboratory, and imaging studies, which complied with the recommendations of the National Comprehensive Cancer Network (NCCN) guidelines for gallbladder cancer (13) and ensured the early detection of potential recurrence. Patients with small cell neuroendocrine carcinoma or a higher K-67 index had a better response to chemotherapy. Recent case reports have demonstrated the efficacy of immunotherapy: nivolumab in an elderly patient (3) and tislelizumab plus chemotherapy achieving complete remission in metastatic GB-NEC (4).

In addition, biological therapy, including somatostatin analogues and interferon, and peptide receptor radionuclide therapy (PRRT) can be effective in somatostatin receptor-positive inoperable gastroenteropancreatic (GEP) neuroendocrine tumors for symptom control (14), and this approach is likely also applicable to inoperable GB-NEC. For GB-NEC patients with distant metastases, systemic therapy and local therapy can often change the prognosis of patients. Local treatment includes radiofrequency ablation (RFA), transarterial chemoembolization (TACE), and selective internal radiation therapy. By controlling liver metastases, it can effectively reduce tumor burden, reduce hormone secretion, and improve the quality of life of patients. Systemic therapy includes biological therapy (such as Somatostatin Analogs (ssA) and octreotide), systemic chemotherapy (such as EP regimen or Epirubicin + Cyclophosphamide (EC) regimen), biological targeting (such as sunitinib and everolimus), and radionuclide therapy (PRRT).

## Conclusion

In summary, GB-NECs are highly malignant and aggressive tumors with non-specific clinical manifestations, and the diagnosis mainly depends on postoperative pathology. Radical resection of gallbladder cancer is the first choice for treatment. Surgical treatment can significantly prolong the survival period of patients, and postoperative chemotherapy can improve the survival period of patients to a certain extent. However, due to the low incidence of the disease, there are few related studies, and there is no generally accepted standardized treatment so far, so further research with a larger sample is still needed.

## Data Availability

The original contributions presented in the study are included in the article/Supplementary Material. Further inquiries can be directed to the corresponding authors.
